# Attention to attention in aphasia – elucidating impairment patterns, modality differences and neural correlates

**DOI:** 10.1016/j.neuropsychologia.2022.108413

**Published:** 2022-11-03

**Authors:** Rahel Schumacher, Ajay D. Halai, Matthew A. Lambon Ralph

**Affiliations:** 1 MRC Cognition and Brain Sciences Unit, University of Cambridge, Cambridge, United Kingdom; 2 Department of Neurology, Inselspital, Bern University Hospital, and University of Bern, Bern, Switzerland

**Keywords:** stroke, aphasia, attention, auditory, visual

## Abstract

It is increasingly acknowledged that patients with aphasia following a left-hemisphere stroke often have difficulties in other cognitive domains. One of these domains is attention, the very fundamental ability to detect, select, and react to the abundance of stimuli present in the environment. Basic and more complex attentional functions are usually distinguished, and a variety of tests has been developed to assess attentional performance at a behavioural level. Attentional performance in aphasia has been investigated previously, but often only one specific task, stimulus modality, or type of measure was considered and usually only group-level analyses or data based on experimental tasks were presented. Also, information on brain-behaviour relationships for this cognitive domain and patient group is scarce.

We report detailed analyses on a comprehensive dataset including patients’ performance on various subtests of two well-known, standardised neuropsychological test batteries assessing attention. These tasks allowed us to explore: 1) how many patients show impaired performance in comparison to normative data, in which tasks and on what measure; 2) how the different tasks and measures relate to each other and to patients’ language abilities; 3) the neural correlates associated with attentional performance.

Up to 32 patients with varying aphasia severity were assessed with subtests from the Test of Attentional Performance (TAP) as well as the Test of Everyday Attention (TEA). Performance was compared to normative data, relationships between attention measures and other background data were explored with principal component analyses and correlations, and brain-behaviour relationships were assessed by means of voxel-based correlational methodology.

Depending on the task and measure, between 3 and 53 percent of the patients showed impaired performance compared to normative data. The highest proportion of impaired performance was noted for complex attention tasks involving auditory stimuli. Patients differed in their patterns of performance and only the performance in the divided attention tests was (weakly) associated with their overall language impairment. Principal components analyses yielded four underlying factors, each being associated with distinct neural correlates.

We thus extend previous research in characterizing different aspects of attentional performance within one sample of patients with chronic post stroke aphasia. Performance on a broad range of attention tasks and measures was variable and largely independent of patients’ language abilities, which underlines the importance of assessing this cognitive domain in aphasic patients. Notably, a considerable proportion of patients showed difficulties with attention allocation to auditory stimuli. The reasons for these potentially modality-specific difficulties are currently not well understood and warrant additional investigations.

## 1 Introduction

Left hemisphere brain lesions often lead to aphasia and, partly due to the pervasiveness of the language disorder, other potentially affected aspects of cognitive functioning are often not considered in detail in this patient group. While the existence of impairments in other cognitive domains as well as their importance for recovery and response to therapy is increasingly acknowledged (Conroy, Drosopoulou, Humphreys, Halai, & Lambon Ralph, 2018; Dignam et al., 2017; El Hachioui et al., 2014; Geranmayeh, Chau, Wise, Leech, & Hampshire, 2017; Glosser & Goodglass, 1990; Helm-Estabrooks, 2002; Lambon Ralph, Snell, Fillingham, Conroy, & Sage, 2010; Murray, 2012; Schumacher, Halai, & Lambon Ralph, 2019; Simic, Rochon, Greco, & Martino, 2019; van de Sandt-Koenderman et al., 2008), detailed investigations regarding impairments in other domains and associated lesion profiles are still scarce.

One of these domains is attention, our ability to detect, select, and react to the abundance of stimuli present in the environment, which is fundamental for nearly all our activities (Corbetta & Shulman, 2002; Duncan, 2006; Petersen & Posner, 2012). Different attentional functions, such as alertness, selective, and divided attention have been described and some frameworks explicitly acknowledge an overlap with the domain of executive functions (e.g., Petersen & Posner, 2012). A variety of tests has been developed to assess attentional performance on a behavioural level (Mirsky, Anthony, Duncan, Ahearn, & Kellam, 1991; Sturm, Willmes, Orgass, & Hartje, 1997; Zimmermann & Leclercq, 2002). While the aspect of accuracy (reacting to a target/distractor stimulus or not) is usually assessed, the aspect of speed (and its variability) is not always measured, despite its importance for activities of daily living (such as driving a car). Also, most tasks (and frameworks) focus on visual (in particular visuospatial) attention and other modalities are less often part of standardised assessments.

On a neuroanatomical level, (visuospatial) attention has typically been related to two main networks, the predominantly right-lateralized ventral attention network, important for bottom-up processing, and the bilateral dorsal attention network, involved in top-down control of attention (Corbetta & Shulman, 2002). A recent proposal, integrating neuroimaging findings and suggesting a common nomenclature for macro-scale networks, called the latter Dorsal Frontoparietal Network and attributed its function to attention, while the former was integrated into the Midcingulo-Insular Network to do with saliency (Uddin, Yeo, & Spreng, 2019). Additionally, the Lateral Frontoparietal Network (Uddin et al., 2019) plays a role in cognitive control and has also been included in an extension of Posner and Petersen’s proposal of three networks for alerting, orienting and executive attention (Petersen & Posner, 2012). Furthermore, task-based functional imaging studies found partially segregated networks for visual versus auditory attention tasks (Braga, Wilson, Sharp, Wise, & Leech, 2013; Salo, Salmela, Salmi, Numminen, & Alho, 2017), indicating that not only general task demands but also stimulus modality might be an important factor to consider when investigating attentional performance and the underlying neural mechanisms.

Attentional impairments are a very common consequence of brain lesions, such as a stroke (Barker-Collo et al., 2009; Hyndman, Pickering, & Ashburn, 2008; Spaccavento et al., 2019). However, much of the patient-related research on attention focused on patients with right hemisphere lesions and on impairments in (visuo)spatial attention allocation. Also, patients with damage to the left hemisphere, in particular patients with aphasia, are often not included in such studies due to their language processing difficulties (Brady, Fredrick, & Williams, 2013). A few studies have specifically investigated aspects of attention in individuals with aphasia (for a review see also Villard & Kiran, 2017). Most of this research focused on the processing of auditory stimuli and reported impairments in basic (Laures, 2005; Shisler Marshall, Basilakos, & Love-Myers, 2013) or more complex auditory attention tests (Kuptsova, Dragoy, & Ivanova, 2021; LaCroix, Baxter, & Rogalsky, 2020) as well as in situations where multiple stimuli must be attended at the same time (Erickson, Goldinger, & LaPointe, 1996). Other recent studies used visual tasks (LaCroix, Tully, & Rogalsky, 2021; Lee, Kocherginsky, & Cherney, 2020) and/or focused on the variabiltiy of reaction times (Naranjo, Grande, & Alted, 2018; Villard & Kiran, 2015, 2018), finding inter-individual differences and some associations with language performance. This latter aspect was also at the core of a study by Murray (2012), showing – by using a range of standardised attention tests – that patients have hetereogenous patterns of impairments and that some attention tests can be useful to explain variance in language-related measures. Taking a slightly different, more data-driven approach to investigate the interrelationship of language, executive functions and some aspects of attention, we recently reported that the variance underlying non-verbal and language test performance was best captured by three orthogonal components each (Shift-Update, Inhibit-Generate, Speed and Phonology, Semantics, Speech Quanta, respectively), all of which were associated with distinct neural clusters (Schumacher et al., 2019).

Taken together, previous research suggests that there are various levels of attentional difficulties in patients with aphasia. However, previous investigations have often only considered one specific task (Erickson et al., 1996; LaCroix et al., 2020; LaCroix et al., 2021; Laures, 2005; Lee et al., 2020; Naranjo et al., 2018), stimulus modality (Erickson et al., 1996; LaCroix et al., 2020; LaCroix et al., 2021; Laures, 2005; Lee et al., 2020; Naranjo et al., 2018; Shisler Marshall et al., 2013), and/or type of measure (Erickson et al., 1996; Shisler Marshall et al., 2013). Also, often only group-level analyses (Erickson et al., 1996; LaCroix et al., 2020; LaCroix et al., 2021; Laures, 2005; Naranjo et al., 2018; Shisler Marshall et al., 2013; Villard & Kiran, 2015, 2018) and/or data based on experimental tasks (Erickson et al., 1996; Laures, 2005; Shisler Marshall et al., 2013; Villard & Kiran, 2015, 2018) were presented. The handful of more thorough investigations including standardised neuropsychological tests either contained very limited information on the aspect of speed (Murray, 2012) or collapsed performance across modalities (Schumacher et al., 2019; Spaccavento et al., 2019). Also, interrelations between measures and the correspondence to proposed models of attention have not been investigated in detail in this patient group. Lastly, the relationships to patients’ lesions were not explored at all or only in a very limited fashion (Kuptsova et al.; LaCroix et al., 2020; Spaccavento et al., 2019).

Here, we report detailed analyses of a comprehensive dataset including patients’ performance on various subtests of two well-known, standardised neuropsychological test batteries assessing attention. The acquired data, ranging from simple to more complex tasks and including visual as well as auditory stimuli, offer accuracy and speed measures. Also, structural neuroimaging was available for all patients, allowing more detailed explorations of brain-behaviour relationships than have previously been reported. Utilising these data, the current study specifically explored: 1) how many patients show impaired performance in comparison to normative data, in which tasks and on what measure; 2) how the different tasks and measures relate to each other and to patients’ language abilities; 3) the neural correlates associated with attentional performance.

## 2 Methods

### 2.1 Participants

This study presents data acquired as part of a project previously reported by our group (Schumacher et al., 2019). In this analysis, we only considered participants for whom we had data on the Alertness, GoNoGo, and Divided Attention subtests from the Test for Attentional Performance (TAP, see below); yielding a sample of 32 (11 female, 21 male; see Table 1 for more details). All participants had a single left-hemispheric stroke (ischaemic or haemorrhagic) at least one year before assessment and imaging, and had no additional significant neurological conditions and no contraindications for MRI. They were pre-morbidly right-handed native English speakers with normal or corrected-to-normal vision. All had been diagnosed with aphasia, but no restrictions were applied regarding the type of aphasia or the severity. Informed consent was obtained from all participants prior to participation, in line with the Declaration of Helsinki and as approved by the local NHS ethics committee.

### 2.2 Neuropsychological Tests

A comprehensive battery of standard neuropsychological tests, assessing simple as well as more complex aspects of attention and including visual as well as auditory stimuli, was chosen for this study. All participants completed the Alertness, GoNoGo, and Divided Attention subtests from the Test for Attentional Performance (Zimmermann & Fimm, 2017), a computerized test battery measuring reaction times and error rates in tests with varying attentional demands. In the Alertness subtest, a cross appears in the centre of a screen at slightly varying time intervals and participants are required to react as rapidly as possible by button press at each appearance. The test measures simple reaction times, which are thought to be an index of ‘tonic’ alertness. The GoNoGo subtest is similar to the Alertness subtest but either a cross or a plus sign appears on the screen for a short duration. Here, participants are required to press the button in response to crosses but withhold any reaction for plus signs. Thus the test assesses the ability to suppress responses to irrelevant stimuli and also determines the choice reaction time under conditions of stimulus selection. The Divided Attention subtest consist of two independent tasks (one visual, one auditory) that must be performed concurrently. In the visual task, a 4x4 array of dots and crosses appears on the screen and participants are asked to press the response button if four crosses form a square. The auditory task consists of high- or low-pitched tones that are played sequentially. A response is required whenever the same tone is played twice consecutively. In all three subtests, quantitative measures (reaction times for correct responses and their standard deviation as a measure of variability), as well qualitative measures (number of omission and commission errors) are collected.

A subset of 26 patients was also administered subtests from the Test of Everyday Attention (Robertson, Ward, Ridgeway, & Nimmo-Smith, 1994). In the Elevator Counting subtest, participants hear strings of tones and must indicate how many tones they counted. In the Elevator Counting with Distraction subtest, the string of tones includes higher pitched distractor tones which are not to be counted. The former is considered to be a test of sustained attention and the latter a test of selective attention (Robertson, Ward, Ridgeway, & Nimmo-Smith, 1996). Participants could indicate their response either by saying the number or by using their fingers/a number strip. Also, before test administration, the sound level was set so that it was easily detectable and comfortable for the individual. Furthermore, it was ensured that participants were able to tell the two different sounds apart (in TAP Divided and TEA Elevator Counting with Distraction).

In addition, comprehensive verbal and nonverbal background testing was available, as reported in previous papers of our group (Butler, Lambon Ralph, & Woollams, 2014; Halai, Woollams, & Lambon Ralph, 2017; Schumacher, Bruehl, Halai, & Lambon Ralph, 2020; Schumacher et al., 2019). The language-based tests included: subtests 1, 2, 8, and 9 from the Psycholinguistic Assessments of Language Processing in Aphasia (PALPA) (Kay, Lesser, & Coltheart, 1992); word-to-picture matching, naming, and Camel and Cactus Test (CCT) from the 64-item Cambridge Semantic Battery (Bozeat, Lambon Ralph, Patterson, Garrard, & Hodges, 2000); the Boston Naming Test (BNT) (Kaplan, Goodglass, & S., 1983); a synonym judgement test (Jefferies, Patterson, Jones, & Lambon Ralph, 2009); the spoken sentence comprehension task from the Comprehensive Aphasia Test (CAT) (Swinburn, Porter, & Howard, 2004); forward and backward digit span (Wechsler, 1987); and the Cookie Theft picture description task from the Boston Diagnostic Aphasia Examination (BDAE) (Goodglass & Kaplan, 1983). The nonverbal tests included: Alertness, GoNoGo, Divided Attention, and Distractibility subtest from the Test of Attentional Performance (TAP) (Zimmermann & Fimm, 2017); the subtests Design Fluency and Trail Making (parts 2–4) from the Delis-Kaplan Executive Function System (D-KEFS) (Delis, Kaplan, & Kramer, 2001); a computerized version of the Tower of London (TOL-F by Schuhfried) (Kaller, Unterrainer, Kaiser, Weisbrod, & Aschenbrenner, 2011); the Kramer test (Balzer et al., 2011); the Raven’s Coloured Progressive Matrices (Raven, 1962); and the Brixton test (Burgess & Shallice, 1997). Performance on these tests served to compute the severity in terms of the breadth of an individual’s language/nonverbal impairment (given as percentage of impaired scores). For example, if a patient’s performance was impaired in nine out of ten administered language tests, their language impairment (or severity) would be given as 90%. Also, patient’s component scores on the three principal language components reported previously (Phonology, Semantics, Speech Quanta; see Schumacher et al., 2019) give an indication of the depth of an individual’s impairments. They were used to elucidate associations between attention and language performance on a more specific level.

### 2.3 Data Analysis

The comprehensive set of administered tests yielded a wealth of measures, allowing a thorough analysis of patients’ attentional performance. Not only reaction times but also standard deviations (as an index of response variability) were considered for all subtests of the TAP. Also, performance on the auditory and visual tasks of the Divided Attention subtest as well as qualitative measures (errors and omissions) were not collapsed but entered separately into the analyses. A subset of the data included here was part of the analyses reported previously (Schumacher et al., 2019).

For each test and measure, patients’ performance was compared to normative data (age-corrected, available for up to the age of 85 for the TAP and 80 for the TEA, so one 88-year-old patient was compared to a slightly younger sample). Performance was considered to be impaired if it was more than 1.5 standard deviations below the mean (i.e., a T-score below 35, a percentile rank below 6 or a scaled score of 5 or lower (Brooks, Sherman, Iverson, Slick, & Strauss, 2011)), except for the TEA Elevator Counting test, where, following the manual, a raw score of 5 or lower was considered as impaired.

To elucidate the commonalities and differences between the various attention tests and measures, and also to test whether the different proposed attentional functions would be captured in this patient sample, principal component analyses (PCA) were computed (using IBM SPSS 27.0) including the TAP measures (replacing the four missing values (two patients without reaction times and respective standard deviations for one subtest) with the mean of the sample). All components with eigenvalues ≥1 were extracted and then varimax rotated, yielding orthogonal and interpretable components. Spearman correlations were computed to elucidate the commonalities between TEA and TAP performance and to investigate the relationship with the severity of patients’ language and nonverbal impairment as well as patient characteristics.

### 2.4 Neuroimaging data acquisition and analysis

High resolution structural T1-weighted Magnetic Resonance Imaging (MRI) scans were acquired on a 3.0 Tesla Philips Achieva scanner (Philips Healthcare, Best, The Netherlands) using an 8-element SENSE head coil. A T1-weighted inversion recovery sequence with 3D acquisition was employed, with the following parameters: TR (repetition time) = 9.0 ms, TE (echo time) = 3.93 ms, flip angle = 8°, 150 contiguous slices, slice thickness = 1 mm, acquired voxel size 1.0 × 1.0× 1.0 mm, matrix size 256 × 256, field of view = 256 mm, TI (inversion time) = 1150 ms, SENSE acceleration factor 2.5, total scan acquisition time = 575 s.

**Figure 1:**
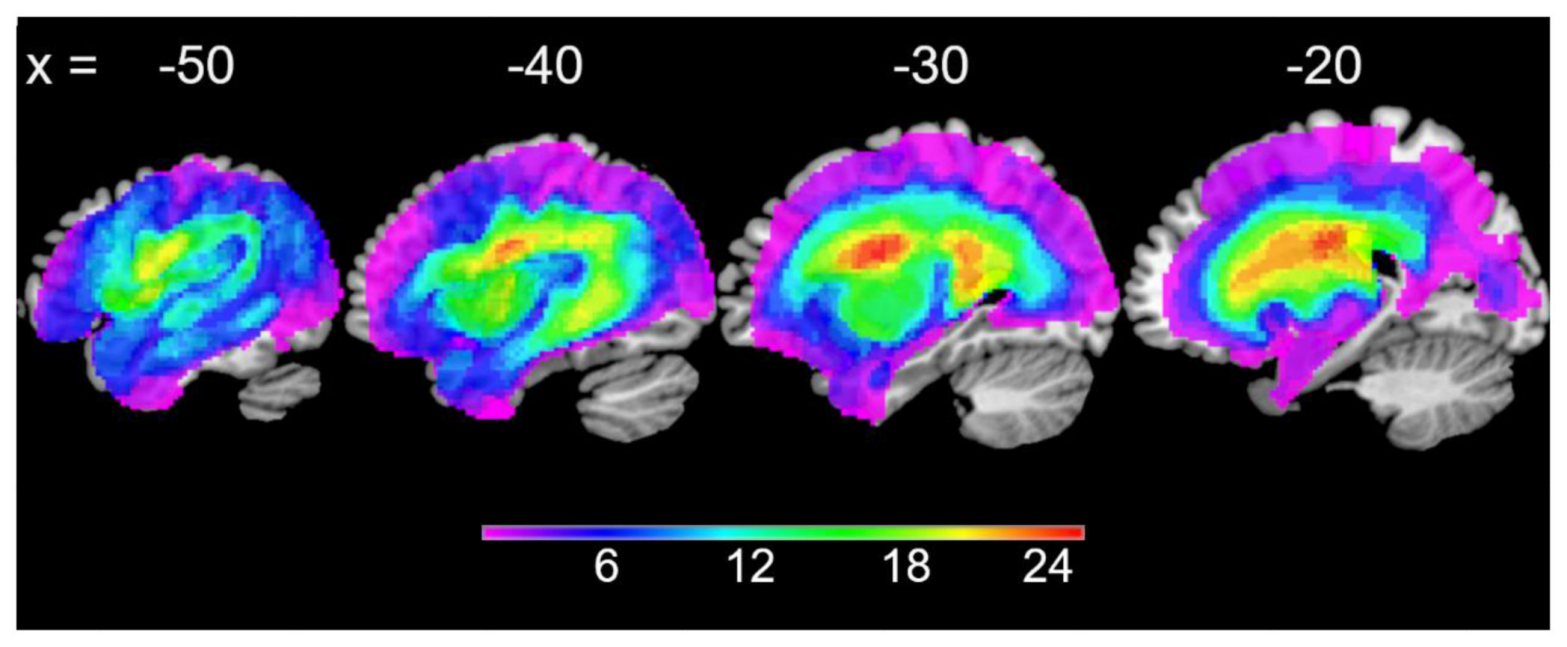
Overlap maps of the patients’ lesions. Corresponding MNI-coordinates are shown above the slices. The figures are thresholded at the maximum overlap (n = 25).

Structural MRI scans were pre-processed with Statistical Parametric Mapping software (SPM8: Wellcome Trust Centre for Neuroimaging, http://www.fil.ion.ucl.ac.uk/spm/). The images were normalised into standard Montreal Neurological Institute (MNI) space using a modified unified segmentation-normalisation procedure optimised for focal lesioned brains (Seghier, Ramlackhansingh, Crinion, Leff, & Price, 2008). Data from all participants with stroke aphasia and from a healthy age and education matched control group (eight female, eleven male) were entered into the segmentation-normalisation. Images were then smoothed with an 8 mm full width- half-maximum (FWHM) Gaussian kernel and used in the lesion analyses described below. The lesion of each patient was automatically identified using an outlier detection algorithm, compared to healthy controls, based on fuzzy clustering. The default parameters were used apart from the lesion definition ‘U-threshold’, which was set to 0.5 to create a binary lesion image. We modified the U-threshold from 0.3 to 0.5 after comparing the results obtained from a sample of patients to what would be nominated as lesioned tissue by an expert neurologist. The images generated were used to create the lesion overlap map in Figure 1.

The normalised and bias-corrected T1-weighted images were used to determine the brain regions where tissue concentration correlated with behaviour using a voxel-based correlational methodology (VBCM) (Tyler, Marslen-Wilson, & Stamatakis, 2005), a variant of voxel-lesion symptom mapping (Bates et al., 2003), in which both the behaviour and signal intensity measures are treated as continuous variables (conducted in SPM12). For the neural correlate analysis, we are assuming that lower T1-weighted intensity is related to tissue damage or atrophy. The participants’ component scores (from the PCA) were entered simultaneously into a VBCM analysis. The resulting lesion clusters thus account for the unique variance of a component. The applied threshold at voxel-level was p <0.001 and at cluster-level p<0.001, unless noted otherwise. The anatomical labels for the clusters were determined using the Harvard–Oxford atlas for grey matter and on the John Hopkins white matter atlas for white matter tracts.

As a methodological aside, we note that tissue integrity across the whole brain is considered in this approach and not just lesion masks from the affected (left, in this case) side. As described previously (Schumacher et al., 2019; Schumacher, Halai, & Lambon Ralph, 2022), the stroke infarctions will be of most importance to explain impaired behavioural performance, but secondary structural changes either following stroke (all our patients were at least 12 months post-stroke), or associated chronic vascular load, or potentially unrelated (for instance age-related) alterations might also play a role. The automatic outlier detection algorithm we used to assess tissue integrity is sensitive enough to capture many of these changes and can therefore yield clusters in the ‘unaffected’ hemisphere.

## 3 Results

### 3.1 Descriptive statistics and comparison to normative data

**Figure 2:**
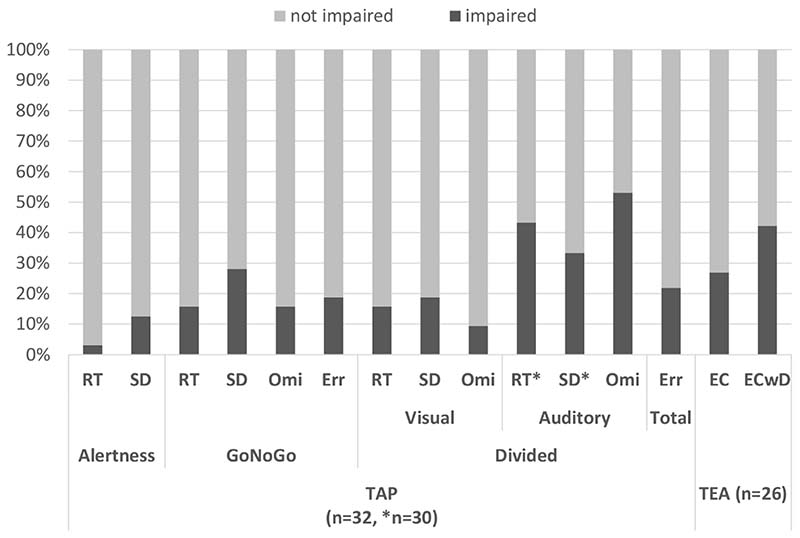
Percentage of patients with impaired performance per task and measure. (TAP = Test for Attentional Performance, TEA = Test of Everyday Attention, RT = reaction time, SD = standard deviation, Omi = omissions, Err = errors, EC = elevator counting, ECwD = EC with Distraction; impairment = lower than -1.5 SD below the mean / raw score of ≤ 5 for EC).

Details on the patients and their performance are summarised in Table 1. As depicted in Figure 2, 3-53 percent of the patients showed impaired performance depending on the task and measure. Performance in the Elevator Counting with Distraction subtest from the TEA as well as the auditory task of the Divided Attention subtest from the TAP were the most commonly compromised; up 50 percent of the patients performed clearly outside normal range in terms of total score or omissions, respectively. In contrast, increased omissions of visual stimuli in the Divided Attention test were only noted in around ten percent of the patients. Considering the reaction times (measured only within the TAP), only one patient’s performance was impaired in the Alertness test, around 15 percent of the patients had abnormally slow reaction times in the GoNoGo subtest and visual task of the Divided Attention subtest, but more than 40 percent showed impaired reaction times to auditory stimuli in the Divided Attention task (the sample size is reduced to 30 as two patients had just one correct reaction to auditory targets). The variability in reacting to targets (standard deviation of correct reactions) was more often impaired than the reaction times (not in case of the auditory task of the Divided Attention subtest) and ranged from 12.5 percent (Alertness) to 33 percent (Divided Attention auditory) of the sample. Also, apart from the omissions in the auditory task of the Divided Attention subtest, all qualitative measures (errors and omissions) were impaired in ten to twenty percent of the sample.

On an individual level, no impaired scores were noted in three patients who completed all tests and in another patient for whom TEA data were not available. In four patients with complete datasets and another one without TEA data, only one measure out of the thirteen/fifteen measures was impaired. Four patients showed impaired scores in more than 50 percent of the measures and, over all the patients and available measures, a quarter of the scores fell in the impaired range.

### 3.2 Underlying attentional components and relationships with background data

**Figure 3:**
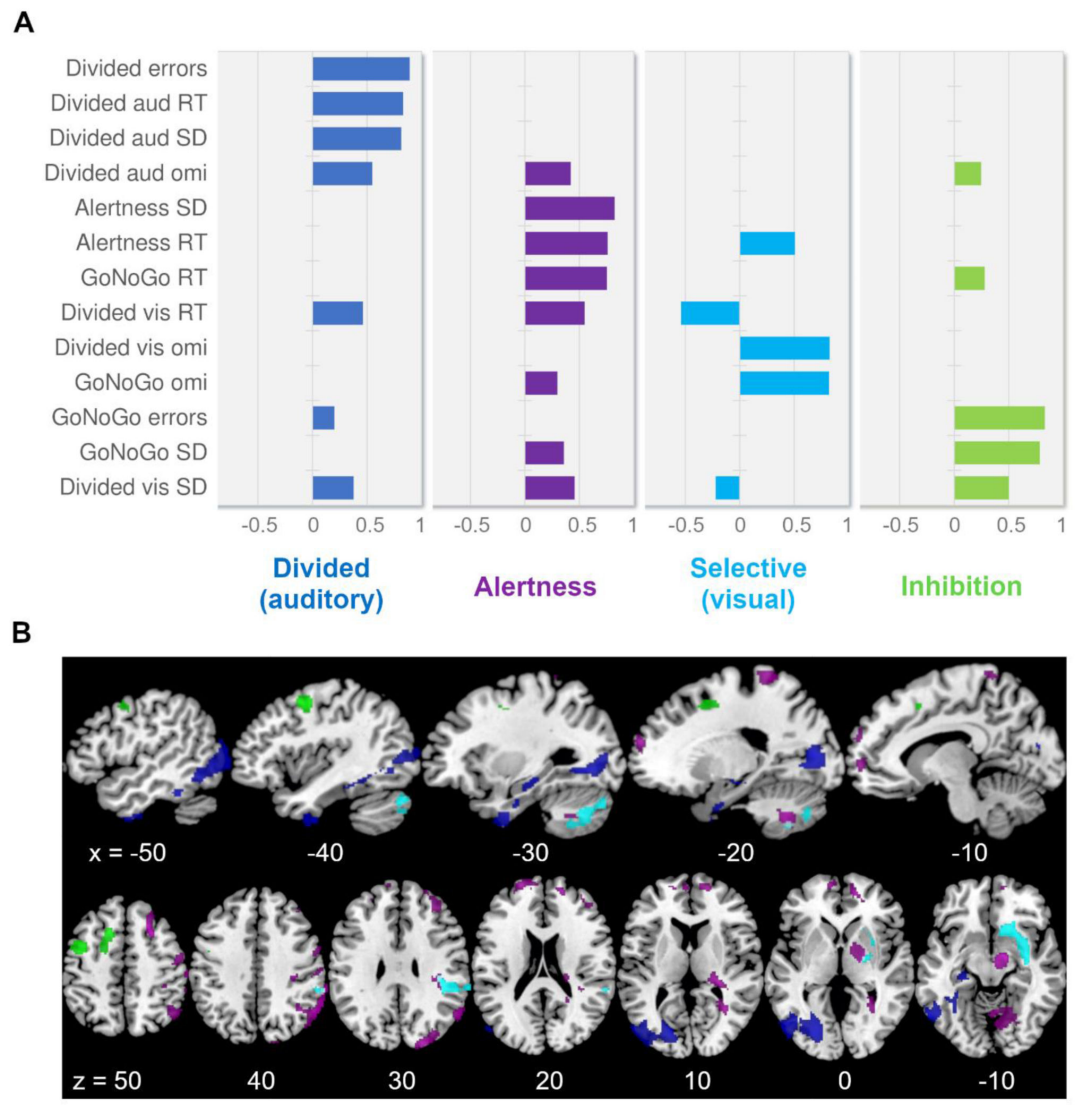
PCA-derived TAP components and their structural correlates. A) The bars represent the loadings of the individual task measures on the components extracted by means of principal components analysis (PCA). The component interpretations are given underneath and the colour-coding is maintained throughout the figure. Loadings < 0.2 are not depicted; aud = auditory; omi = omissions; RT = reaction time; SD = standard deviation; vis = visual. B) Voxel-based correlational methodology clusters were obtained by applying a voxel-level threshold of p ≤ 0.001), and a family-wise error correction of p ≤ 0.001 on cluster-level (p ≤ 0.002 for the Inhibition component). The slices are indicated in MNI-space and in neurological convention (left is left).

Next, to elucidate commonalities between the different attention tests and measures, a PCA was performed on the TAP data and correlations with TEA performance were computed. The PCA on the TAP data (KMO = 0.618, Bartlett’s test of sphericity p < 0.001) revealed four components, accounting for 72.7% of the variance. The loadings of all TAP measures on the four components are shown in Figure 3A. The first component explained 32.7% and was interpreted as capturing (auditory) Divided Attention because all measures of the auditory task of the Divided Attention subtest plus the errors in that test loaded highly on this component. The second component explained 17.8% and was interpreted as capturing Alertness because the measures of this test, alongside the reaction time in the GoNoGo test loaded highest on this component. The third component explained 12.3% and seems to capture (visual) selective attention with omissions in the GoNoGo test and the visual task of the Divided Attention subtest loading highest. Lastly, the fourth component explained 9.9% of the variance and might be interpreted as capturing Inhibition as its highest loadings are the error rate and standard deviation of the GoNoGo test.

To avoid a reduction of the sample size, TEA measures were not included in the PCA but correlations with the derived factor scores were computed to elucidate the relationships among these attentional measures (a table showing all correlations between raw test measures is included in the Supplementary Material). As shown in Table 2, the Elevator Counting task was not significantly associated with any component of the TAP. In turn, performance on the Elevator Counting with Distraction task was significantly correlated with the factor score of the (auditory) Divided Attention component as well as with the Inhibition component.

Table 2 also shows how the derived TAP components and the TEA measures relate to the severity of patients’ nonverbal and language impairment, to the three principal language components (Phonology, Semantics, Speech Quanta) as well as to patient characteristics. Of note here is that only TEA Elevator Counting with Distraction was associated with the severity of patients’ language (as well as the nonverbal) impairment. No significant association with patients’ overall language impairment was found for the TEA Elevator Counting or any of the TAP components. At the level of individual TAP measures, only two scores were significantly associated with patients’ language impairment, the visual and auditory omissions in the Divided Attention subtest (r_s_(30) = .459, p < 0.01; r_s_(30) = .392, p < 0.05, respectively). Similarly, we found very limited associations between attention measures and the three principal language components. Only Speech Quanta was significantly, albeit moderately and not consistently, associated with two TAP components. More detailed information on correlations with language performance is provided in the Supplementary Material. Lastly, with respect to patient characteristics, significant associations were only found for the first TAP component (age, time post-stroke, lesion volume).

These analyses thus not only confirm that different attentional aspects are differently affected across patients, but also that processing auditory stimuli in more complex situations seems to form an independent behavioural component which mirrors the findings from our normative comparisons.

### 3.3 Lesion correlates

To investigate the associations between patients’ lesions and their performance in the attention tests, we performed a VBCM analysis by simultaneously including an individual’s scores on the four PCA-derived components as continuous variables, thus yielding clusters that explain variance uniquely associated with each component. Significant clusters emerged for all components, as depicted in Figure 3B and detailed in Table 3. The first component, mainly reflecting performance in the auditory subtest of the Divided Attention test was associated with a left-lateralised cluster mainly including inferior lateral temporooccipital areas. The second component, predominantly capturing performance in the Alertness test was associated with a number of smaller bilaterally scattered clusters, the majority being in right frontoparietal regions. The third component, reflecting omissions of visual stimuli, was again associated with right hemisphere regions, one cluster included the putamen, insula and frontal orbital cortex, while the other cluster was located in parietal cortex and included the supramarginal gyrus. The second and third component were also associated with bilateral cerebellar clusters. Lastly, the fourth component, reflecting response variability and errors in the GoNoGo test, was associated with left frontal regions, in particular the superior and middle left frontal gyrus. Importantly, a control analysis including age and lesion volume as covariates of no interest yielded very similar clusters for all components.

## 4 Discussion

To gain a better understanding of attentional impairments in patients with chronic stroke aphasia, a comprehensive dataset including patients’ performance on various subtests of two well-known, standardised neuropsychological test batteries assessing attention was analysed and related to patients’ background data as well as brain lesions. Patients differed markedly in their patterns of performance and the highest proportion of impairments was noted for complex attention tasks involving auditory stimuli. These were also the only tasks where a significant (albeit rather weak) association with patients’ language impairment could be established. Going beyond the correlational analyses reported in previous research, we show that attentional performance was captured by four independent components, all of which were associated with distinct structural correlates.

### 4.1 Prevalence of attentional impairments

Patients’ performance varied greatly depending on the task and measure, which replicates previous accounts of differentially impaired aspects of attentional function. While the aspect of intensity (Alertness) was only rarely affected, performance was impaired in around a quarter of the sample when the selectivity aspect was taxed, and half of the patients showed difficulties in more complex auditory or divided attention tasks. This aligns with the theoretical notion of there being a hierarchy among the different attentional aspects with intensity forming the foundation, followed by selectivity in single-, then dual-task conditions (Sturm et al., 1997). The patterns also roughly correspond to previous investigations (Murray, 2012; Spaccavento et al., 2019), but direct comparisons of our TEA data with Murray (2012) are complicated by the use of a different cut-off for defining impaired performance. In comparison to Spaccavento et al. (2019) who employed the same tasks (or very similar versions) of the TAP and the same cut-off to determine impaired performance, we found a smaller proportion of impaired performance for the Alertness and GoNoGo test than they reported for their subsample of patients with left hemisphere stroke (around 30% and 45%, respectively). The most probable explanation for this difference is that they also included patients in the subacute stage post stroke where patients usually present with more severe impairments. Relatedly, it is important to point out that the analyses presented here probably underestimate the prevalence of attentional impairments, particularly for the more basic aspects of attention. This is because only patients who had complete datasets for the Alertness, GoNoGo and Divided Attention tests were included in the analyses. Based on clinical experience, more severely affected patients – not only with respect to basic aspects of attention but also other cognitive functions – sometimes struggle with the task demands of more complex selective and divided attention tasks (see also Spaccavento et al., 2019). Accordingly, these more severe patients would be less likely to appear in our data analysis yet carry a higher probability of showing impaired performance in the more basic tasks.

From a clinical perspective, it is also noteworthy that the variability of performance (in terms of the standard deviations of the correct reactions) seems to be a more sensitive measure of impairment than the reaction times themselves. For all tasks except the auditory subtest of the Divided Attention, the percentage of impaired scores was higher for the standard deviations than the respective reaction times. Increased response variability following acquired brain injury has been noted previously in aphasia (Naranjo et al., 2018; Villard & Kiran, 2015, 2018) and in stroke more generally (Shalev, Humphreys, & Demeyere, 2016). However, it remains to be further elucidated whether it can be considered a proxy of sustained attention or a less specific marker of performance (Shalev et al., 2016) and to what extent it is useful for predicting recovery and levels of functioning.

### 4.2 Relationships among attention measures and beyond

Extending previous research, we compared performance in various attention tests, many of which yield more than one measure. We investigated the components underlying performance in three TAP subtests and found four factors that explained nearly three quarters of the variance. The first component reflects performance in Divided Attention (in particular the auditory part of the task), the second Alertness (or speed, more generally), the third (visual) Selective Attention, and the fourth Inhibition.

While this investigation focused on attention tests and included a range of time- as well as accuracy-based measures in the visual and auditory modality, the findings mirror our previous study (Schumacher et al., 2019) which had a much broader approach but included fewer measures of attention (e.g., no measures of variability, visual and auditory measures not separate). More specifically, the Divided Attention component is similar to Shift-Update, Alertness to Speed and Inhibition to Inhibit-Generate (Schumacher et al., 2019). Also, comparable components have been derived from healthy participants’ performance on the TAP battery (Zimmermann & Fimm, 2017). Thus, the measures obtained from the Alertness, GoNoGo and Divided Attention TAP subtests seem to capture three important and independent building blocks of cognitive functioning in health and disease. Essentially, even if termed ‘tests of attention’, some of the measures tap the space where attention and executive functions overlap. Somewhat relatedly, we would like to note that no tests specifically assessing spatial attention were included in our study. It has been shown that spatial attention allocation can also be affected in patients with left hemisphere lesions (e.g., Blini et al., 2016) and it remains to be elucidated in which way the underlying factor structure would change if this aspect of attention was included.

As for the relationship between the TAP components and the two tests from another neuropsychological battery assessing attention, the TEA, our analyses reveal two main findings. First, the Elevator Counting with Distraction (conceptualised as a test of selective attention), was most strongly associated with the Inhibition component but also with (auditory) Divided Attention. While the first association seems to reflect the fact that only some sounds must be counted while others are to be ignored (similar to the visual GoNoGo), the second association probably captures the auditory nature of the task. Fittingly, in a factor analysis including all TEA tasks alongside other cognitive measures, Elevator Counting with Distraction loaded highest on a factor termed ‘auditory-verbal working memory’ (Robertson et al., 1996). Second, however, the simpler Elevator Counting task seems to capture something different, as no significant associations with the TAP components were found. The main reason for this lack of association with other measurements (see also below) is probably that many of the patients performed at ceiling. Interestingly, one other study employing the TEA in a slightly bigger sample of patients with aphasia (Murray, 2012), reported significant correlations between Elevator Counting and non-verbal cognitive tests as well as with aphasia severity. In our study, Elevator Counting with Distraction was significantly correlated with aphasia severity but this was not the case for Elevator Counting, nor for any of the TAP attention components. The reasons for these differences between studies are unclear but could have to do with the patient sample (potentially less severely affected in our study) or with the index used to indicate aphasia severity. Overall – in line with our previous findings (Schumacher et al., 2019) and with other researchers (e.g., Villard & Kiran, 2018) – we found only very limited cross-correlations between patients’ performance in attention and language tests in this study.

In sum, our behavioural findings underscore that attention is multi-factorial and that patients with aphasia may show different patterns of attentional impairments that are largely independent of the severity of their language impairment. Given the documented influence of non-verbal cognition on aphasia therapy and outcome (Conroy et al., 2018; Dignam et al., 2017; El Hachioui et al., 2014; Fillingham, Sage, & Lambon Ralph, 2005; Geranmayeh et al., 2017; Lambon Ralph et al., 2010; Simic et al., 2019; van de Sandt-Koenderman et al., 2008), but also the relevance of targeting specific attentional functions during therapy (Sturm et al., 1997), it is paramount to not only assess this aspect of cognitive functioning in sufficient breadth and depth in patients with aphasia, but to offer this patient group targeted therapeutic interventions also beyond language.

### 4.3 Modality-specific difficulties?

The strikingly worse performance in tasks with auditory stimuli was somewhat surprising. However, it appears to be in line with one previous study that also assessed attention in tasks of varying complexity and with visual and auditory stimuli (Villard & Kiran, 2015). That study reported slower and less accurate responses, at the group-level, to auditory stimuli in more complex tasks. In other previous investigations reporting difficulties in tests with auditory stimuli (Erickson et al., 1996; LaCroix et al., 2021; Laures, 2005; Shisler Marshall et al., 2013), only responses to auditory stimuli were collected, hence no comparison between auditory and other sensory modalities can be made. Critically, poorer performance when reacting to auditory compared to visual stimuli might be attributable to differences in task difficulty or other task characteristics that are confounded with the modality. More specifically, in this study, the auditory subtest of the Divided Attention test requires binding over time (comparing the just presented tone with the previous one in order to decide whether they were the same or not) and therefore poses higher demands on working memory than the visual subtest, where binding in space is required.

Beyond specific tasks demands, other reasons for putative modality-specific difficulties are conceivable. First, low performance in attention tests with auditory stimuli as well as in language tests may be caused by difficulties in more basic auditory processing, as for instance shown in Wernicke’s patients (Robson, Griffiths, Grube, & Woollams, 2019; Robson, Grube, Lambon Ralph, Griffiths, & Sage, 2013). Importantly, and in contrast to the corresponding parts in the visual system, primary and secondary auditory cortex is located in superior temporal regions and therefore part of the same medial cerebral artery vascular territory that is commonly associated with post-stroke aphasia. These regions were indeed lesioned in some, but not all, of the patients performing worst in the auditory task of the Divided Attention subtest. Second, moving beyond primary/secondary areas, recent neuroimaging research in neurotypical controls revealed that brain regions and connections involved in higher-order information processing also seem to be organised in a modality-biased way (Assem, Shashidhara, Glasser, & Duncan, 2021; Braga, Hellyer, Wise, & Leech, 2017; Mayer, Ryman, Hanlon, Dodd, & Ling, 2017; Noyce, Cestero, Michalka, Shinn-Cunningham, & Somers, 2017; Salo et al., 2017). Thus it is conceivable that, even if such modality-biased regions are small and appear to be interdigitated, some patients’ lesions may affect more of the auditory-biased parts/connections, leading to auditory attention (and possibly language processing) difficulties. Indeed, the patients performing worst in the auditory task of the Divided Attention subtest but without evident damage to the primary/secondary auditory cortices, had damage to frontal and/or subcortical regions. Third, and somewhat relatedly, a visual bias in cognition has been described (Posner, Nissen, & Klein, 1976; Spence, 2009) and is gaining renewed interest in the context of these most recent neuroimaging findings (e.g., Assem et al., 2021; Mayer et al., 2017). Interestingly, modality-dominance patterns seem to change with age and the observed stronger visual dominance in older adults was explained with an increased imbalance of intersensory inhibition (Barnhart, Rivera, & Robinson, 2018). Similar mechanisms might underlie a potentially enhanced visual dominance following a stroke. Lastly, and a limitation of our study, there was no formal standardised assessment of patients’ hearing. Even if unlikely, given that the sound level was always adjusted to be at an individually comfortable level and task comprehension was ensured by means of practice trials, this factor cannot be ruled out completely.

In sum, while our findings, close examination of previous reports, as well as recent neuroscientific findings hint at the possibility of at least some patients showing modality-specific difficulties with auditory stimuli, it remains to be investigated if and for which patients this is the case and why. An increased understanding of potential modality-specific versus modality-independent or supramodal attention allocation difficulties is not only of theoretical but also of clinical relevance. Current therapies for aphasia are for instance often based on audio-visual stimulation, an approach that might not be the most suitable in all circumstances. Also, a better understanding of the mechanisms underlying patients’ difficulties is pivotal not only for tailoring therapeutic approaches in the rehabilitation setting, but also for adapting eventual compensatory strategies for these patients in daily life.

### 4.4 Structural correlates of attentional performance

Previous research has only rarely associated patients’ performance in attention tests with their underlying brain pathology and we are not aware of any detailed investigation of this sort in patients with aphasia. Here, we elucidated these brain-behaviour relationships by means of voxel-based correlational methodology. We found significant and distinct clusters for all four PCA-derived TAP components. Two of the components (Divided (auditory) and Inhibition) were associated with - posterior and anterior, respectively - left hemisphere clusters. The other two components (Alertness and Selective (visual)) were (predominantly) associated with right-sided fronto-parietal as well as bilateral cerebellar clusters. As noted above, there was a close correspondence between the behavioural components derived from an extensive set of attentional measures alone (presented here) compared to analyses including limited information on attentional performance but instead additional measures of executive functions and language (Schumacher et al., 2019). These similarities are also reflected in the neural clusters associated with the attention components.

The Divided (auditory) component cluster, comprising posterior lateral temporo-occipital areas, overlaps considerably with the Shift-Update cluster reported previously (Schumacher et al., 2019). Based on the tasks loading on the underlying behavioural component in the previous study, we had interpreted this cluster location as relating to visuo-spatial task demands (Fedorenko, Duncan, & Kanwisher, 2013; Humphreys & Lambon Ralph, 2017; Simpson et al., 2011). However, given that the behavioural component in the current analysis mainly reflects performance in a non-spatial auditory task (performed concurrently with a visuo-spatial task), such an interpretation does not go far enough. Indeed, even if not always specifically mentioned, inferior lateral temporo-occipital cortex regions are commonly reported as being part of the Dorsal Frontoparietal Network (or Dorsal Attention Network) as well as, more anteriorly, the Lateral Frontoparietal Network (or Multiple-Demand Network) (Assem, Glasser, Van Essen, & Duncan, 2020; Gordon et al., 2017; Uddin et al., 2019). Crucially, it is not only found when contrasting hard and easy tasks in the visual but also in the auditory modality (Assem et al., 2021). Moreover, not only the cortical regions but also their white matter connections are crucial. Prefrontal regions, mentioned above in relation to putative modality-specific difficulties, might seem far away but are connected to these lateral occipito-temporal regions, for instance via the (potentially lesioned) inferior fronto-occipital fasciculus.

The Inhibition component, reflecting commission errors in the GoNoGo task, was exclusively associated with left frontal regions. In addition to partially overlapping with our previous Inhibit-Generate cluster (Schumacher et al., 2019) and with aspects of patients’ performance in verbally based tests of executive function (Schumacher et al., 2022), the finding aligns with numerous studies assigning the dorsolateral prefrontal cortex an important role in control and selection (e.g., Assem et al., 2020; Uddin et al., 2019).

The closest correspondence between the current and the previously reported (Schumacher et al., 2019) clusters was found for Alertness which is perhaps least surprising, given the overlap between the behavioural measures included in the Alertness vs. Speed components. The associated clusters included a number of smaller, mainly right-sided, fronto-parietal regions. This finding is in keeping with previous research suggesting that the right hemisphere (and brain stem structures) play a dominant role in regulating (phasic) alertness (Petersen & Posner, 2012; Spaccavento et al., 2019; Sturm & Willmes, 2001).

One behavioural component, Selective (visual), had no equivalent in our previous work. The clusters associated with this component overlapped in part with brain regions found in our previous analyses (right superior parietal and orbitofrontal) for the Inhibit-Generate as well as Speed components. The present analyses also showed an association with the right putamen, a brain region that did not appear as clearly previously. The putamen has been identified as an important convergence zone mediating between attentional and control networks (Jarbo & Verstynen, 2015). As such, an association with omissions in attentional tasks requiring selection might not be altogether surprising, even if it has been associated with perseverations in patients with neglect following a right-hemisphere stroke (Kaufmann et al., 2018).

Taken together, the brain-behaviour analysis shows that all four behavioural components were associated with distinct neural correlates. Our findings not only provide important converging evidence from a patient group that is understudied in this respect but also reveal potentially interesting avenues for future research, for instance regarding the functional role of the left inferior temporo-occipital region.

### 4.5 Conclusion

We extended previous research in characterizing different aspects of attentional performance within a sample of patients with chronic post stroke aphasia. Our behavioural findings underscore the multi-factorial and language-independent patterns of attentional impairments these patients can have and which should be specifically diagnosed and treated. Notably, a considerable proportion of patients showed difficulties with complex attention tasks including auditory stimuli. The reasons for these potentially modality-specific difficulties are currently not well understood and warrant additional investigations. Lastly, our brain-behaviour analysis, which yielded distinct clusters underlying all four behavioural components, provides important converging evidence from a patient group that is understudied in this respect.

## Supplementary Material

Supplementary Material

## Figures and Tables

**Table 1:** Patient characteristics and descriptive statistics of performance in attention tests.

	Mean	SD	Range
Age (years)	61.6	11.4	45 - 88
Education (years)	12.6	2.7	9 - 19
Time post-stroke (years)	6.5	4.8	2 - 24
Lesion volume (voxels)	15074	10978	175 - 37907
Verbal impairment (%)	60.9	21.8	21.4 - 100
Nonverbal impairment (%)	32.0	15.7	6.3 - 70
TAP Alertness Median RT	248.0	42.8	194 - 399
TAP Alertness SD	48.2	42.3	16.5 - 220.7
TAP GoNoGo Median RT	487.4	80.4	371 - 720
TAP GoNoGo SD	113.4	50.3	29.4 - 250.8
TAP GoNoGo Errors	3.2	3.1	0 - 14
TAP GoNoGo Omissions	1.0	3.0	0 - 15
TAP Divided auditory Omissions	4.0	4.8	0 - 15
TAP Divided auditory Median RT	788.5	327.4	495 - 2024
TAP Divided auditory SD	240.3	191.4	69.3 - 867.9
TAP Divided visual Omissions	2.8	3.0	0 - 15
TAP Divided visual Median RT	889.5	141.6	437 - 1217
TAP Divided visual SD	287.0	119.3	82.8 - 506
TAP Divided Errors	4.2	4.1	0 - 15
TEA Elevator Counting score	5.7	1.8	0 - 7
TEA Elevator Counting with Distraction score	4.2	3.3	0 - 10

Note: Attention measures statistics are based on raw scores. RT = reaction time, SD = standard deviation

**Table 2:** Spearman correlations among attentional measures and with impairment severity, language components as well as patient characteristics.

		TEA		TAP components			
		EC	ECwD	Divided (auditory)	Alertness	Selective (visual)	Inhibition
**TEA**	EC		.087	-.236	-.346	.316	-.233
	ECwD			**-.404** *	-.137	-.183	-.473*
**Severity**	Nonverbal	.002	**-.585** **	.417*	.173	.296	.333
	Verbal	.158	**-.500** **	.262	-.062	.166	.159
**Language components**	Phonology	.029	.169	-.074	-.040	.004	.161
	Semantics	-.206	.203	-.254	-.203	.018	-.023
	Speech Quanta	-.129	.366	-.238	**.372** *	**-.387** *	-.253
**Patient characteristics**	Age	-.037	-.369	**.494** **	.221	.175	.272
	Education	-.009	.127	-.211	.150	-.148	-.180
	Time post	-.257	-.355	**.471** **	-.016	-.096	.266
	Lesion	.143	-.097	**.418** *	-.137	.202	.063

Note:

* p< 0.05 two-tailed,

** p< 0.01 two-tailed; n = 26 for correlations including TEA measures, else n = 32. Correlations between TAP derived components and nonverbal severity are greyed out because some TAP measures were used to calculate severity. Higher scores on the TAP components and severity indicate worse performance whereas higher scores on the language components and TEA indicate better performance. EC = Elevator Counting, ECwD = Elevator Counting with Distractor

**Table 3:** Clusters and peaks associated with the TAP component scores.

Component	Extent	Location	L/R	Z	x	y	z
**Divided (auditory)**	2602	Inferior temporal gyrus post	L	5.13	-58	-44	-24
		Forceps major	L	4.40	-20	-78	10
		Lateral occipital cortex inf	L	4.39	-58	-62	-10
	474	Parahippocampal gyrus ant	L	4.17	-30	-10	-30
		Parahippocampal gyrus ant	L	4.08	-26	-4	-38
		Inferior temporal gyrus ant	L	3.96	-46	-6	-46
**Alertness**	1109	Superior frontal gyrus	R	6.08	26	-2	60
	577	Superior longitudinal fas	R	5.67	42	-26	32
	483	Thalamus	R	5.48	12	-6	-10
	330	Postcentral gyrus	R	5.26	44	-36	60
	281	Occipital pole	R	5.17	22	-90	32
	303	Postcentral gyrus	L	5.13	-14	-46	74
	614	Frontal Pole	R	5.04	18	56	18
	433	Corticospinal tract	R	4.93	32	-58	-40
	729	Lateral occipital cortex sup	R	4.93	52	-60	42
	316	Cerebellum	L	4.85	-18	-58	-44
	347	Frontal Pole	L	4.70	-22	62	20
	855	Inferior frontal occipital fas	R	4.65	34	-56	10
**Selective (visual)**	654	Cerebellum	L	5.96	-34	-78	-34
	404	Parietal operculum cortex	R	4.94	50	-32	28
	1027	Frontal orbital cortex	R	4.79	22	24	-14
	351	Cerebellum	R	4.36	30	-70	-36
**Inhibition**	264	Middle frontal gyrus	L	4.14	-40	0	50
	295	Superior frontal gyrus	L	3.99	-14	16	50
		Superior frontal gyrus	L	3.45	-24	2	48
		Middle frontal gyrus	L	3.15	-34	4	44

Note: For components associated with more than two clusters, only the first peak per cluster is included in the table. Coordinates are in MNI space. L/R = left or right side of the brain, ant = anterior, fas = fasciculus, inf = inferior, p ope = pars opercularis, p tri = pars triangularis, post = posterior, sup = superior

## References

[R1] Assem M, Glasser MF, Van Essen DC, Duncan J (2020). A Domain-General Cognitive Core Defined in Multimodally Parcellated Human Cortex. Cerebral Cortex.

[R2] Assem M, Shashidhara S, Glasser MF, Duncan J (2021). Precise Topology of Adjacent Domain-General and Sensory-Biased Regions in the Human Brain. Cerebral cortex (New York, NY: 1991).

[R3] Balzer C, Berger JM, Caprez G, Gonser A, Gutbrod K, Keller M (2011). Rheinfelden.

[R4] Barker-Collo SL, Feigin VL, Lawes CMM, Parag V, Senior H, Rodgers A (2009). Reducing Attention Deficits After Stroke Using Attention Process Training. Stroke.

[R5] Barnhart WR, Rivera S, Robinson CW (2018). Different patterns of modality dominance across development. Acta Psychologica.

[R6] Bates E, Wilson SM, Saygin AP, Dick F, Sereno MI, Knight RT, Dronkers NF (2003). Voxel-based lesion–symptom mapping. Nature Neuroscience.

[R7] Blini E, Romeo Z, Spironelli C, Pitteri M, Meneghello F, Bonato M, Zorzi M (2016). Multi-tasking uncovers right spatial neglect and extinction in chronic left-hemisphere stroke patients. Neuropsychologia.

[R8] Bozeat S, Lambon Ralph MA, Patterson K, Garrard P, Hodges JR (2000). Non-verbal semantic impairment in semantic dementia. Neuropsychologia.

[R9] Brady MC, Fredrick A, Williams B (2013). People with aphasia: capacity to consent, research participation and intervention inequalities. International Journal of Stroke.

[R10] Braga RM, Hellyer PJ, Wise RJ, Leech R (2017). Auditory and visual connectivity gradients in frontoparietal cortex. Human Brain Mapping.

[R11] Braga RM, Wilson LR, Sharp DJ, Wise RJS, Leech R (2013). Separable networks for top-down attention to auditory non-spatial and visuospatial modalities. Neuroimage.

[R12] Brooks BL, Sherman EMS, Iverson GL, Slick DJ, Strauss E, Schoenberg MR, Scott JG (2011). Little Black Book of Neuropsychology: a Syndrome-Based Approach.

[R13] Burgess P, Shallice T (1997). The Hayling and Brixton tests.

[R14] Butler RA, Lambon Ralph MA, Woollams AM (2014). Capturing multidimensionality in stroke aphasia: mapping principal behavioural components to neural structures. Brain.

[R15] Conroy P, Drosopoulou CS, Humphreys GF, Halai AD, Lambon Ralph MA (2018). Time for a quick word? The striking benefits of training speed and accuracy of word retrieval in post-stroke aphasia. Brain.

[R16] Corbetta M, Shulman GL (2002). Control of goal-directed and stimulus-driven attention in the brain. Nature Reviews Neuroscience.

[R17] Delis DC, Kaplan E, Kramer JH (2001). Delis-Kaplan Executive function system: examiners manual.

[R18] Dignam J, Copland D, O'Brien K, Burfein P, Khan A, Rodriguez AD (2017). Influence of Cognitive Ability on Therapy Outcomes for Anomia in Adults With Chronic Poststroke Aphasia. Journal of Speech Language and Hearing Research.

[R19] Duncan J (2006). Brain mechanisms of attention. Quarterly Journal of Experimental Psychology.

[R20] El Hachioui H, Visch-Brink EG, Lingsma HF, van de Sandt-Koenderman MWME, Dippel DWJ, Koudstaal PJ, Middelkoop HAM (2014). Nonlinguistic Cognitive Impairment in Poststroke Aphasia: A Prospective Study. Neurorehabilitation and Neural Repair.

[R21] Erickson RJ, Goldinger SD, LaPointe LL (1996). Auditory vigilance in aphasic individuals: Detecting nonlinguistic stimuli with full or divided attention. Brain and Cognition.

[R22] Fedorenko E, Duncan J, Kanwisher N (2013). Broad domain generality in focal regions of frontal and parietal cortex. Proceedings of the National Academy of Sciences of the United States of America.

[R23] Fillingham JK, Sage K, Lambon Ralph MA (2005). Treatment of anomia using errorless versus errorful learning: are frontal executive skills and feedback important?. International Journal of Language & Communication Disorders.

[R24] Geranmayeh F, Chau T, Wise RJS, Leech R, Hampshire A (2017). Domain-general subregions of the medial prefrontal cortex contribute to recovery of language after stroke. Brain.

[R25] Glosser G, Goodglass H (1990). Disorders in executive control functions among aphasic and other brain-damaged patients. Journal of Clinical and Experimental Neuropsychology.

[R26] Goodglass H, Kaplan E (1983). The assessment of aphasia and related disorders.

[R27] Gordon EM, Laumann TO, Gilmore AW, Newbold DJ, Greene DJ, Berg JJ, Dosenbach NUF (2017). Precision Functional Mapping of Individual Human Brains. Neuron.

[R28] Halai AD, Woollams AM, Lambon Ralph MA (2017). Using principal component analysis to capture individual differences within a unified neuropsychological model of chronic post-stroke aphasia: Revealing the unique neural correlates of speech fluency, phonology and semantics. Cortex.

[R29] Helm-Estabrooks N (2002). Cognition and aphasia: a discussion and a study. Journal of Communication Disorders.

[R30] Humphreys GF, Lambon Ralph MA (2017). Mapping Domain-Selective and Counterpointed Domain-General Higher Cognitive Functions in the Lateral Parietal Cortex: Evidence from fMRI Comparisons of Difficulty-Varying Semantic Versus Visuo-Spatial Tasks, and Functional Connectivity Analyses. Cerebral Cortex.

[R31] Hyndman D, Pickering RM, Ashburn A (2008). The influence of attention deficits on functional recovery post stroke during the first 12 months after discharge from hospital. Journal of Neurology, Neurosurgery & Psychiatry.

[R32] Jarbo K, Verstynen TD (2015). Converging Structural and Functional Connectivity of Orbitofrontal, Dorsolateral Prefrontal, and Posterior Parietal Cortex in the Human Striatum. The Journal of Neuroscience.

[R33] Jefferies E, Patterson K, Jones RW, Lambon Ralph MA (2009). Comprehension of Concrete and Abstract Words in Semantic Dementia. Neuropsychology.

[R34] Kaller CP, Unterrainer JM, Kaiser S, Weisbrod M, Aschenbrenner S (2011). Moedling.

[R35] Kaplan E, Goodglass HSW (1983). The Boston Naming Test.

[R36] Kaufmann BC, Frey J, Pflugshaupt T, Wyss P, Paladini RE, Vanbellingen T, Nyffeler T (2018). The spatial distribution of perseverations in neglect patients during a nonverbal fluency task depends on the integrity of the right putamen. Neuropsychologia.

[R37] Kay J, Lesser R, Coltheart M (1992). PALPA: Psycholinguistic Assessments of Language Processing in Aphasia.

[R38] Kuptsova SV, Dragoy OV, Ivanova MV (2021). Switching attention deficits in post-stroke individuals with different aphasia types. Aphasiology.

[R39] LaCroix AN, Baxter LC, Rogalsky C (2020). Auditory attention following a left hemisphere stroke: comparisons of alerting, orienting, and executive control performance using an auditory Attention Network Test. Auditory perception & cognition.

[R40] LaCroix AN, Tully M, Rogalsky C (2021). Assessment of alerting, orienting, and executive control in persons with aphasia using the Attention Network Test. Aphasiology.

[R41] Lambon Ralph MA, Snell C, Fillingham JK, Conroy P, Sage K (2010). Predicting the outcome of anomia therapy for people with aphasia post CVA: both language and cognitive status are key predictors. Neuropsychological rehabilitation.

[R42] Laures JS (2005). Reaction time and accuracy in individuals with aphasia during auditory vigilance tasks. Brain and Language.

[R43] Lee JB, Kocherginsky M, Cherney LR (2020). Attention in individuals with aphasia: Performance on the Conners' Continuous Performance Test-2nd edition. Neuropsychological Rehabilitation.

[R44] Mayer AR, Ryman SG, Hanlon FM, Dodd AB, Ling JM (2017). Look hear! The prefrontal cortex is stratified by modality of sensory input during multisensory cognitive control. Cerebral Cortex.

[R45] Mirsky AF, Anthony BJ, Duncan CC, Ahearn MB, Kellam SG (1991). Analysis of the elements of attention: A neuropsychological approach. Neuropsychology review.

[R46] Murray LL (2012). Attention and Other Cognitive Deficits in Aphasia: Presence and Relation to Language and Communication Measures. American Journal of Speech-Language Pathology.

[R47] Naranjo NP, Grande DD, Alted CG (2018). Individual variability in attention and language performance in aphasia: a study using Conner's Continuous Performance Test. Aphasiology.

[R48] Noyce AL, Cestero N, Michalka SW, Shinn-Cunningham BG, Somers DC (2017). Sensory-biased and multiple-demand processing in human lateral frontal cortex. Journal of Neuroscience.

[R49] Petersen SE, Posner MI, Hyman SE (2012). Annual Review of Neuroscience.

[R50] Posner MI, Nissen MJ, Klein RM (1976). VISUAL DOMINANCE - INFORMATION-PROCESSING ACCOUNT OF ITS ORIGINS AND SIGNIFICANCE. Psychological Review.

[R51] Raven JC (1962). Coloured Progressive Matrices, Sets A, AB, B.

[R52] Robertson IH, Ward T, Ridgeway V, Nimmo-Smith I (1994). The test of everyday attention (TEA).

[R53] Robertson IH, Ward T, Ridgeway V, Nimmo-Smith I (1996). The structure of normal human attention: The Test of Everyday Attention. Journal of the International Neuropsychological Society.

[R54] Robson H, Griffiths TD, Grube M, Woollams AM (2019). Auditory, Phonological, and Semantic Factors in the Recovery From Wernicke’s Aphasia Poststroke: Predictive Value and Implications for Rehabilitation. Neurorehabilitation and Neural Repair.

[R55] Robson H, Grube M, Lambon Ralph MA, Griffiths TD, Sage K (2013). Fundamental deficits of auditory perception in Wernicke's aphasia. Cortex.

[R56] Salo E, Salmela V, Salmi J, Numminen J, Alho K (2017). Brain activity associated with selective attention, divided attention and distraction. Brain research.

[R57] Schumacher R, Bruehl S, Halai AD, Lambon Ralph MA (2020). The verbal, non-verbal and structural bases of functional communication abilities in aphasia. Brain Communications.

[R58] Schumacher R, Halai AD, Lambon Ralph MA (2019). Assessing and mapping language, attention and executive multidimensional deficits in stroke aphasia. Brain.

[R59] Schumacher R, Halai AD, Lambon Ralph MA (2022). Assessing executive functions in post-stroke aphasia—utility of verbally based tests. Brain Communications.

[R60] Seghier ML, Ramlackhansingh A, Crinion J, Leff AP, Price CJ (2008). Lesion identification using unified segmentation-normalisation models and fuzzy clustering. Neuroimage.

[R61] Shalev N, Humphreys G, Demeyere N (2016). Assessing the temporal aspects of attention and its correlates in aging and chronic stroke patients. Neuropsychologia.

[R62] Shisler Marshall R, Basilakos A, Love-Myers K (2013). Further Evidence of Auditory Extinction in Aphasia. Journal of Speech, Language, and Hearing Research.

[R63] Simic T, Rochon E, Greco E, Martino R (2019). Baseline executive control ability and its relationship to language therapy improvements in post-stroke aphasia: a systematic review. Neuropsychological Rehabilitation.

[R64] Simpson GV, Weber DL, Dale CL, Pantazis D, Bressler SL, Leahy RM, Luks TL (2011). Dynamic Activation of Frontal, Parietal, and Sensory Regions Underlying Anticipatory Visual Spatial Attention. Journal of Neuroscience.

[R65] Spaccavento S, Marinelli CV, Nardulli R, Macchitella L, Bivona U, Piccardi L, Angelelli P (2019). Attention deficits in stroke patients: The role of lesion characteristics, time from stroke, and concomitant neuropsychological deficits. Behavioural neurology.

[R66] Spence C, Srinivasan N (2009). Progress in Brain Research.

[R67] Sturm W, Willmes K (2001). On the Functional Neuroanatomy of Intrinsic and Phasic Alertness. NeuroImage.

[R68] Sturm W, Willmes K, Orgass B, Hartje W (1997). Do specific attention deficits need specific training?. Neuropsychological Rehabilitation.

[R69] Swinburn K, Porter G, Howard D (2004). Comprehensive aphasia test.

[R70] Tyler LK, Marslen-Wilson W, Stamatakis EA (2005). Dissociating neuro-cognitive component processes: voxel-based correlational methodology. Neuropsychologia.

[R71] Uddin LQ, Yeo BTT, Spreng RN (2019). Towards a Universal Taxonomy of Macro-scale Functional Human Brain Networks. Brain Topography.

[R72] van de Sandt-Koenderman WME, van Harskamp F, Duivenvoorden HJ, Remerie SC, van der Voort-Klees YA, Wielaert SM, Visch-Brink EG (2008). MAAS (Multi-axial Aphasia System): realistic goal setting in aphasia rehabilitation. International Journal of Rehabilitation Research.

[R73] Villard S, Kiran S (2015). Between-session intra-individual variability in sustained, selective, and integrational non-linguistic attention in aphasia. Neuropsychologia.

[R74] Villard S, Kiran S (2017). To what extent does attention underlie language in aphasia?. Aphasiology.

[R75] Villard S, Kiran S (2018). Between-session and within-session intra-individual variability in attention in aphasia. Neuropsychologia.

[R76] Wechsler DA (1987). Wechsler Memory Scale - Revised Manual.

[R77] Zimmermann P, Fimm B (2017). TAP-M Test of Attentional Performance (Mobility Version): Psytest.

[R78] Zimmermann P, Leclercq M, Leclercq M, Zimmermann P (2002). Applied Neuropsychology of Attention: Theory, Diagnosis and Rehabilitation.

