# Supplementary material for: Attention to attention in aphasia – elucidating impairment patterns, modality differences and neural correlates
Source: Neuropsychologia. Author manuscript; Available in PMC 2023 Apr 19. (PMC7614452; doi:10.1016/j.neuropsychologia.2022.108413)
Supplement: Supplementary Material [file EMS173950-supplement-Supplementary_Material.pdf]

Attention to attention in aphasia – elucidating impairment patterns, modality differences and neural correlates

Rahel Schumacher, Ajay D. Halai, Matthew A. Lambon Ralph

SUPPLEMENTARY MATERIAL

Table 1. Patient characteristics.

Subject	Age	Sex	Edu-cation	years post-stroke	lesion volume	BDAE classification	verbal impairment[#]	nonverbal impairment[#]
1	55	m	17	9	11915	Broca	57.1	37.5
2	55	f	12	12	9767	Anomia	28.6	12.5
3	71	m	11	8	8788	MNF	42.9	43.8
4	61	m	11	17	18392	Broca	50.0	31.3
5	47	m	11	6	8437	Anomia	28.6	31.3
6	50	f	11	8	6975	Anomia	64.3	43.8
7	79	f	11	7	13577	Anomia	64.3	70.0
8	63	f	19	6	9159	Anomia	42.9	6.3
9	80	m	13	5	34242	MNF	71.4	43.8
10	71	m	11	4	3311	Anomia	50.0	18.8
11	62	m	11	5	16433	Anomia	50.0	18.8
12	70	m	13	7	33239	Global	100.0	31.3
13	52	m	13	8	22948	Anomia	78.6	31.3
14	48	f	16	4	3897	Conduction	71.4	37.5
15	46	f	13	5	18948	Anomia	57.1	18.8
16	75	f	11	6	23863	TMA	57.1	31.3
17	76	f	11	15	12057	MNF	85.7	68.8
18	45	f	16	3	175	Anomia	21.4	25.0
19	66	m	11	4	33239	MNF	85.7	50.0
20	69	m	11	5	31317	MNF	92.9	37.5
21	47	m	11	3	10409	Anomia	42.9	31.3
22	59	f	11	24	12699	Anomia	71.4	37.5
23	68	m	11	2	4879	Conduction	50.0	18.8
24	53	m	11	7	37822	Global	100.0	25.0
25	88	m	9	2	8528	Anomia	57.1	41.7
26	67	m	17	2	6557	Conduction	64.3	31.3
27	57	m	16	2	6974	Anomia	57.1	12.5
28	66	m	10	7	6607	Anomia	50.0	6.3
29	50	m	19	2	4538	Anomia	35.7	6.3
30	51	m	11	3	14681	Anomia	35.7	37.5
31	56	f	11	2	10081	MNF	100.0	50.0
32	69	m	12	7	37907	Broca/ MNF	85.7	50.0

Notes: f = female, m= male, MNF = Mixed Nonfluent, # impairment = percentage of impaired test scores (more than 1.5 standard deviations below the mean of controls), based on available tests per participant

Table 2. Spearman correlations between all raw attention measures.

	TEA		TAP												
			**Alertness**		**Go/NoGo**				**Divided**						
	**EC (1)**	**ECwD (2)**	**Med (3)**	**SD (4)**	**Med (5)**	**SD (6)**	**Err (7)**	**Omi (8)**	**Med-a (9)**	**SD-a (10)**	**Omi-a (11)**	**Med-v (12)**	**SD-v (13)**	**Omi-v (14)**	**Err (15)**
**(1)**		.087	-.073	-.174	-.095	-.208	-.058	.011	.002	-.136	-.124	-.184	-.536**	.144	-.279
**(2)**			-.136	-.310	-.259	-.577**	-.475*	-.296	-.334	-.395	-.578**	-.282	-.443*	-.490*	-.444*
**(3)**				.683**	.301	.125	-.045	.328	.321	.084	.075	.190	.270	.302	-.067
**(4)**					.237	.239	.190	.378*	.485**	.342	.243	.430*	.348	.454**	.178
**(5)**						.606**	.054	.170	.218	.300	.392*	.226	.361*	.228	.011
**(6)**							.486**	.401*	.257	.463**	.543**	.247	.542**	.245	.205
**(7)**								.228	.276	.220	.189	.098	.255	.183	.187
**(8)**									.255	.134	.284	-.049	.282	.531**	.126
**(9)**										.582**	.195	.278	.269	.398*	.415*
**(10)**											.370*	.554**	.467**	.269	.568**
**(11)**												.380*	.434*	.340	.392*
**(12)**													.544**	.197	.443*
**(13)**														.274	.597**
**(14)**															.348

Notes: * p< 0.05, ** p< 0.01 two-tailed; n = 32 for TAP, n = 26 for TEA; EC = Elevator Counting, wD = with Distraction, Med = Median, SD = Standard Deviation, Omi = Omissions, Err = Errors, a = auditory, v = visual

Table 3. Spearman correlations between performance on language tests and attention, language as well as severity measures.

	**TEA**		**TAP components**				**Language components**			**Severity**	
	**EC**	**ECwD**	**Divided (auditory)**	**Alertness**	**Selective (visual)**	**Inhibition**	**Phonology**	**Semantics**	**Speech Quanta**	**Verbal**	**Non-verbal**
**Digit span forward**	.049	-.006	-.048	.005	.114	.207	.883**	.154	.028	-.640**	-.232
**Digit span backward**	.101	.283	-.142	.157	-.093	-.038	.783**	-.060	.433*	-.767**	-.340
**Repetition**	-.108	.431*	-.197	.011	-.069	-.119	.801**	.336	.248	-.808**	-.417*
**Naming**	-.120	.258	-.179	-.063	-.052	-.111	.799**	.357*	.258	-.788**	-.341
**Minimal Pairs**	.041	.364	-.089	-.149	-.186	-.028	.616**	.220	-.016	-.636**	-.327
**Camel & Cactus pictures**	-.229	.300	-.267	-.082	-.161	-.187	.094	.874**	.317	-.557**	-.423*
**Synonym judgment**	.060	.268	-.316	-.029	.052	-.329	.280	.615**	.462**	-.609**	-.481**
**Word-picture matching**	-.004	.293	-.280	-.073	-.192	.072	.517**	.556**	.249	-.733**	-.515**
**Spoken comprehension**	-.036	.360	-.420*	-.176	.078	-.055	.622**	.545**	.302	-.746**	-.608**
**Cookie Theft Token**	-.063	.439*	-.179	.327	-.293	-.271	.090	.205	.899**	-.406*	-.357*
**Cookie Theft MLU**	-.052	.489*	-.293	.254	-.233	-.257	.257	.255	.895**	-.584**	-.459**
**Cookie Theft WPM**	-.185	.419*	-.162	.414*	-.436*	-.235	.334	.135	.824**	-.680**	-.422*

Notes: * $p < 0.05$, ** $p < 0.01$ two-tailed; n = 26 for correlations including TEA measures, else n = 32; EC = Elevator Counting, wD = with Distraction, MLU = mean length of utterance, WPM = words per minute
